# Anaesthesiology students’ Non-Technical skills: development and evaluation of a behavioural marker system for students (AS-NTS)

**DOI:** 10.1186/s12909-019-1609-8

**Published:** 2019-06-13

**Authors:** Parisa Moll-Khosrawi, Anne Kamphausen, Wolfgang Hampe, Leonie Schulte-Uentrop, Stefan Zimmermann, Jens Christian Kubitz

**Affiliations:** 1 Department of Anaesthesiology, University Medical Center Hamburg-Eppendorf, Martinistr. 52, 20246 Hamburg, Germany; 2 Institute of Biochemistry and Molecular Cell Biology, University Medical Center Hamburg-Eppendorf, Martinistr. 52, 20246 Hamburg, Germany

**Keywords:** Non-technical skills, Simulation, Education

## Abstract

**Background:**

Non-Technical Skills (NTS) are becoming more important in medical education. A lack of NTS was identified as a major reason for unsafe patient care, favouring adverse events and team breakdown. Therefore, the training of NTS should already be implemented in undergraduate teaching. The goal of our study was to develop and validate the Anaesthesiology Students’ Non-Technical Skills (AS-NTS) as a feasible rating tool to assess students’ NTS in emergency and anaesthesiology education.

**Methods:**

The development of AS-NTS was empirically grounded in expert- and focus groups, field observations and data from NTS in medical fields. Validation, reliability and usability testing was conducted in 98 simulation scenarios, during emergency and anaesthesiology training sessions.

**Results:**

AS-NTS showed an excellent interrater reliability (mean 0.89), achieved excellent content validity indexes (at least 0.8) and was rated as feasible and applicable by educators. Additionally, we could rule out the influence of the raters’ anaesthesiology and emergency training and experience in education on the application of the rating tool.

**Conclusions:**

AS-NTS provides a structured approach to the assessment of NTS in undergraduates, providing accurate feedback. The findings of usability, validity and reliability indicate that AS-NTS can be used by anaesthesiologists in different year of postgraduate training, even with little experience in medical education.

**Electronic supplementary material:**

The online version of this article (10.1186/s12909-019-1609-8) contains supplementary material, which is available to authorized users.

## Background

In patient care, both Technical Skills (TS) and Non-Technical Skills (NTS) are necessary to maintain best practice as well as reach a high level of expertise [1]. TS are routinely taught in trainee programs. However, evaluation and assessment of NTS have been missing for a long time [2, 3]. NTS are defined as “the cognitive, social and personal resource skills that complement technical skills and contribute to safe and efficient task performance” [4].

Adverse events in high-risk settings often take place due to deficiencies in NTS [5], which has been shown in various fields such as aviation and nuclear energy [6–8] and has also been confirmed for medical care: up to 70% of adverse events are due to human errors [9–11]. In order to reduce medical errors, good NTS and improved teamwork are essential [12].

NTS interventions and mostly feedback on NTS have shown to have positive effects on team performance, concluding that good patient care requires TS and NTS, which have been found to correlate in crew resource management [13, 14] and to foster improved clinical performance like quicker problem solving in simulated operating theatre environment [15]. The positive effects of NTS also encompass enhanced patient safety. Salas et al. showed in a meta-analysis the positive effects of NTS on team members’ reactions and attitudes related to teamwork safety [16]. Other studies pointed out positive effects of NTS on clinical performance (TS) and on patient outcome like surgical complications and morbidity [17].

Knowledge of necessity and benefits of NTS lead many institutions to emphasize the importance of interpersonal skills [13, 16–21] and these departments implemented crew-resource training programs in their curricula, primarily focussing on NTS during postgraduate training [22].

The training of NTS should not only be focused in postgraduate training - undergraduate curricula and education should already integrate the concept of “patient safety”, directly addressing, teaching and assessing NTS [23, 24]. Only a few studies have investigated the effect of teaching NTS in undergraduates. Hagemann et al. showed that even one brief seminar had positive effects on undergraduates’ NTS [21].

The German Association for Medical Education has acknowledged the repeatedly expressed need of NTS implementation in undergraduate education by publishing a “Learning Objective Catalogue for Patient Safety in Undergraduate Medical Education”, which has the aim to unify the curricular targets in German medical faculties [25]. However, a concrete curriculum, implementation or teaching strategy for NTS in undergraduate education is not given yet. In addition, due to a missing conventional rating tool a structured assessment of NTS in undergraduates is often lacking.

To create and realize an implementation and teaching strategy for NTS in undergraduates, at first the structured assessment of NTS with a robust method is necessary [4], in order to provide specific and formative feedback and to monitor the learning progress in undergraduates.

Several rating tools for assessing NTS in medical professionals are available. They are helpful to provide feedback which is not based on “gut feeling” and to speak “the same language” during the feedback process [26–31]. However, the existing rating tools, such as the *Anaesthetists’ Non-Technical Skills* (ANTS) [32], are very complex and not designed for undergraduates or junior residents, as ANTS is developed for experienced anaesthesiologists to rate trainees who have reached certain TS, which limits its broad application in undergraduate education. A feasible application is further limited as for raters a two-day training with the rating scheme is required. The use of ANTS delays the feedback loop, as the NTS ratings are based on video clips of the training sessions, which are evaluated after the training [5].

The goal of this study was to develop a rating tool to assess NTS in undergraduate education in emergency medicine and anaesthesiology: Anaesthesiology Students’Non-Technical Skills (AS-NTS). The tool is supposed to be feasible and easily handled without the necessity for video recording or extended instructions and trainings for the user.

## Methods

### Study design

This study was performed at the Department of Anaesthesiology in the University Medical Center Hamburg-Eppendorf, Germany. The study was conducted in the period of Janurary 2017 to December 2017. Undergraduates and residents in anaesthesiology participated in this study with a stepwise design in order to develop and validate a rating tool for NTS in undergraduates in anaesthesiology. The development took place in four steps (Table 1), empirically grounded on qualitative and quantitative research methods:

1. Review of published literature (expert group)
2. Focus group and half-structured interviews
3. Field observation
4. Implementation and validation

**Table 1:** Development steps of AS-NTS

Qualitative and quantitative research methodology			Conducted Steps
Development			
Development step	1.	Expert group^a^	• Literature search • Development of a NTS list and discussion of their relevance for undergraduates
	2.	Focus groups^b^ and half-structured interviews	• Discussion of NTS • Development of a hierarchical structure of NTS
Validation			
Development step	3.	Field Observation	• Testing of feasibility and practicality • Evaluation if the skills were observable
	4.	a) Evaluation questionnaire b) Analyzing the interrater reliability	• Calculation of the content validity index^c^ • Validation of usability and feasibility • Assessment of NTS in 98 simulation scenarios during emergency training sessions by two/ three independent raters

Legend: ^**a**^ The expert group consisted of two anaesthesiology specialists with profound experience in medical education and of two specialists who work on the assessment of psycho-social skills in Multiple Mini Interviews for the purpose of student selection, one of them being a psychologist and one of them highly qualified in medical education. The composition of the expert group was chosen to combine expertise of anaesthesiology, medical education and psychology

^b^The focus group consisted of five anaesthesiology specialists, two male and three female participants, with a median age of 34.8 years. The mother language of all participants is German and they all had completed their residency at the Department of Anaesthesiology, University Medical Center Hamburg Eppendorf. The qualification criteria to join the focus group were 1) to be at anaesthesiology specialist level and 2) to work regularly in anaesthesiology and emergency medicine and in medical teaching. Routine in emergency care was required in order to link the theoretical discussion about the NTS with reality settings

^c^The content validity index for each dimension was calculated, reflecting the proportion of relevance [33]

Table 1 shows a scheme of the conducted developmental steps and underlying research methods.

A detailed explanation of the development is given in the Additional file 1.

### Study setting: assessment of NTS during emergency and anaesthesiology training sessions

The undergraduate curriculum of the Medical Faculty of Hamburg has implemented emergency training sessions in nearly every semester, in order to experience the students in emergency medicine. We use high fidelity simulators (Rescue Anne Laerdal) which are suitable for training technical skills such as endotracheal intubation, defibrillation or drug administration.

NTS were assessed in four different training sessions (Advanced cardiac life support I, II, III and operation room simulation) of four different semesters. In each training session a pre-existing set of standardized simulation scenarios were used *(13 in total, a detailed description of the simulation scenarios is provided in the* Additional file 1).

The simulation scenarios are standardised and solely for each type of training session. For example, the training session “Advanced cardiac life support II (ACLS II)”, which is held in the 3rd year of undergraduate education, includes the scenarios: “*Hyperkalaemia”, “Hypothermia” and “Aspiration”.*

In each training session every student is assigned to a small group which rotates through each simulation scenario.

With each following semester, the simulation scenarios require more advanced TS and NTS. In order to rule out that low NTS skills are due to technical skills being not proceduralized we decided to test our rating system in students who had already passed the basic life support training in following training sessions:

- **○ Advanced cardiac life support I** *(ACLS I: 2nd or 3rd year undergraduates, pre-existing simulation scenarios: 2; number of rated simulation scenarios for interrater agreement analysis: 20)*
- **○ Advanced cardiac life support II** (ACLS II: 3rd year undergraduates, *pre-existing simulation scenarios: 3; number of rated simulation scenarios for interrater agreement analysis: 24)*
- **○ Advanced cardiac life support III** (ACLS III: 4th year undergraduates, *pre-existing simulation scenarios: 5; number of rated simulation scenarios for interrater agreement analysis: 23)*
- **○ Operation room (OR) simulation** (3rd or 4th year undergraduates, *pre-existing simulation scenarios: 3; number of rated simulation scenarios for interrater agreement analysis: 31)*

In each training session, the undergraduates are divided into groups of three. Each of these groups rotates through the simulation scenarios of the training session. In each simulation scenario one student takes the role of the physician, the other two that of paramedics or anaesthetic co-workers. The student in the role of the physician leads the team and delegates basic tasks such as establishing the monitoring, preparing defibrillation and other required medical procedures to the other team members. Therefore, only this student was evaluated by the two or three supervising anaesthesiologists, using the AS-NTS.

### Raters and interrater reliability

Twenty-one anaesthesiologists (Table 2) conducted the training sessions during the study period. In 67 emergency simulation scenarios two of them rated the students independently, in the 31 operating room simulation scenarios three raters were involved. The raters who rated the same simulation scenario, did not discuss their results while rating, in order to rule out cognitive bias.

**Table 2:** Characteristics of the twenty-one raters

Sex	Mean Age	Experience in medical Education	Anaesthesiology training
Female: 13	31.7 years	High*1: 5	Attendings: 5
Male:8		Medium*2: 8	5th year residents: 5
		Low*3:8	4th year residents: 4
			3rd year residents: 4
			2nd year residents: 3

Legend: *1: High experience: organization of and high involvement in undergraduate teaching *2: Medium experience: certain routine in undergraduate teaching *3: Low experience: introduced to undergraduate teaching during the study time

The rater teams changed frequently based on the teaching schedule. The raters all received a five-minute introduction into the AS-NTS.

The interrater-reliability was investigated using a two-step approach.

In the first step a classical analysis of interrater-reliability was conducted, analysing data from rating pairs. To rule out agreement by chance, the intraclass correlation (ICC) from six pairs of raters were calculated, which had rated at least six simulation scenarios together. The first analysis included 67 of the total of 98 simulation scenarios. In five of the six pairs the first author (R1) took part (Table 3).

**Table 3:** Characteristics of the six pairs of raters

Pairings	Sex	Age	Experience in medical education	Anaesthesiology training	Number of rated simulation scenarios
R1/R2	F/M	32/27	High/Low	A./2nd year resident	17
R1/R3	F/M	32/27	High/Middle	A./3rd year resident	14
R1/R4	F/F	32/34	High/High	A./5th year resident	7
R1/R5	F/M	32/38	High/Middle	A./A.	8
R1/R6	F/F	32/29	High/Low	A./4th year resident	14
R7/R8	M/F	31/28	Middle/Low	5th−/3rd year resident	7

Abbreviation: *A* Attending, *R* Rater

In the second step of the interrater-reliability analysis, the whole data set from the 98 simulation scenarios was analysed. To rule out that either the strong involvement of R1 in the development process of AS-NTS or the medical training had an effect on the interrater reliability, data was aggregated across raters being in the same year of training. This allowed us to investigate the relationship between medical expertise and rating agreement (Table 4).

**Table 4:** Ratings and comparisons by anaesthesiology training after data aggregation

Year of anaesthesiology training	Number of rated simulation scenarios
Attending (*n* = 5) vs 2nd year resident (*n* = 3)	23
Attending (*n* = 5) vs 3rd year resident (*n* = 4)	16
Attending (*n* = 5) vs 4th year resident (*n* = 4)	20
Attending (*n* = 5) vs 5th year resident (*n* = 5)	11
4th year (*n* = 4) vs 3rd year resident (*n* = 4)	15
5th year (*n* = 5) vs 3rd year resident(*n* = 4)	13

### Statistical analysis

Statistical analysis was performed using IBM SPSS Statistics Version 23.0. Intraclass correlation (ICC) was used for ordinally scaled data and Cohens Kappa for nominally scaled data to calculate interrater reliability. We used the *one-way random effects model* to calculate the ICCs [34]. Values of ICC and kappa below 0.40 are interpreted as poor correlation, between 0.40 and 0.59 as fair correlation, between 0.60 and 0.74 as good correlation and between 0.75 and 1.00 as excellent correlation [35].

## Results

### Development of the AS-NTS assessment tool

The literature search resulted in 12 different NTS important in anaesthesiology (Table 5). The discussions in the focus- and expert group revealed, that not all of these NTS are highly important for undergraduates. During the field observations some NTS were difficult to observe. Using the results of the focus group discussions we defined new dimensions specifically for undergraduates, symbiosing some pre-defined NTS:

- Planing tasks, prioritising and conducting
- Teamwork: exchanging information and leading the team
- Team orientation

**Table 5:** Hierarchical mapping of Non-Technical skills and multi-step development of AS-NTS

Non-Technical Skill	Step 1: Literature	Step 2: Focus group		Step 3: Field Observation		Part of rating tools		
		Highly important	Important	Skill (precursor skill) Observable	Skill difficult to observe	ANTS system	AS-NTS	
Situational awareness	[26, 28, 30, 36–45]		*		*	*		
Prioritising (Planning Tasks)	[12, 31, 46]	*		*		*	*	DIMENSION 1: Planning tasks, prioritising and problem solving
Decision-making	[12, 28, 41, 42, 47–54]	*		(*)		*	*	
Maintaining standards	[31, 38, 39, 43, 54]		*	(*)		*	*	
Coordinating team members and activities	[39, 55]	*		*		*	*	DIMENSION 2: Teamwork and leadership
Communication	[12, 22, 26, 28–31, 38, 40, 41, 43–47, 49–51, 53–59]	*		*			*	
Leadership	[7, 12, 16, 26, 29–31, 36, 39, 41–44, 46, 47, 55–58]	*		*			*	
Using authority and assertiveness	[38, 39, 56]		*	(*)		*	*	
Team-building	[12, 28, 31, 36, 38, 39, 56]		*	*			*	DIMENSION 3: Team orientation
Team orientation/ Teamwork	[12, 28, 29, 31, 36, 38, 39, 45, 46, 56–58]	*		*		*	*	
Resolving conflicts/ problems	[12, 39, 42, 47, 56]		*		*			
Supporting others	[22, 28, 39, 42]		*		*	*		

**Fig. 1:**
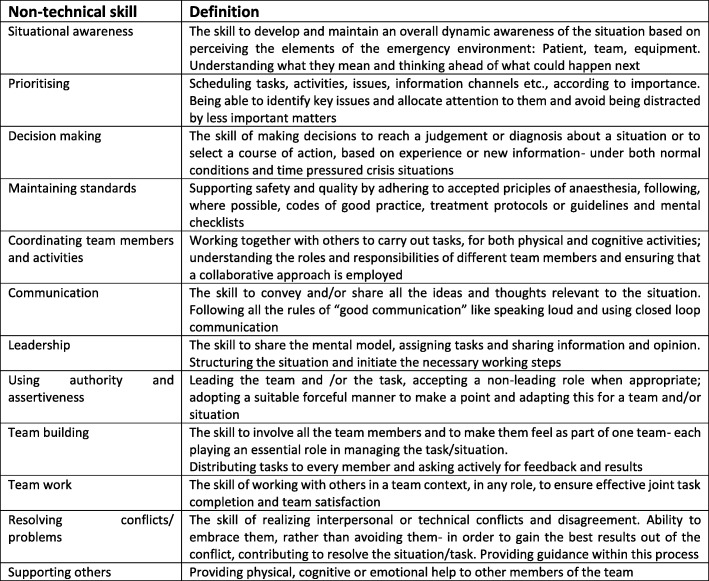
Definition of the NTS. The definitions were extracted from the cited taxonomies in Table 5, mostly the ANTS system

Table 5 displays the created list of the NTS and the further conducted steps which were decisive for the inclusion of each skill. The last column illustrates which NTS is part of ANTS and AS-NTS. Figure 1 displays the definition of the NTS.

**Fig. 2:**
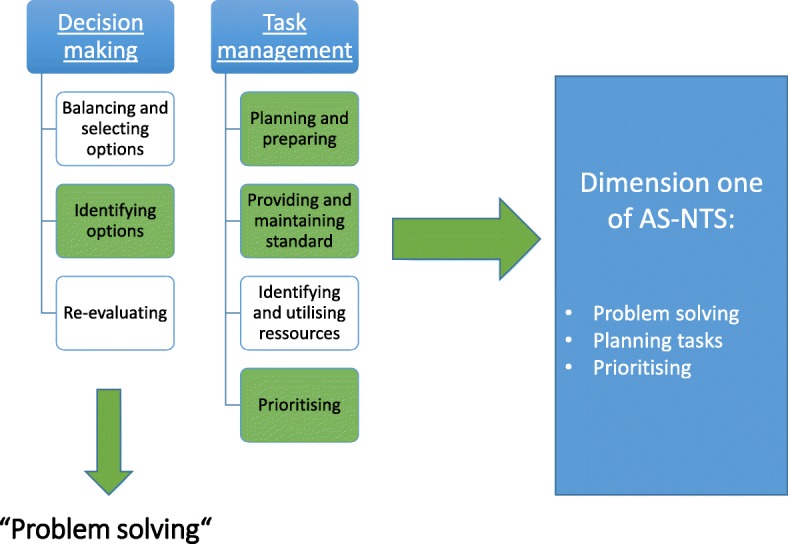
Underlying NTS for dimension one of AS-NTS

The first dimension of AS-NTS:

*“Planning tasks, prioritizing and problem solving”* resulted as a compound, mainly formed by pre-defined NTS dimensions “Decision making” and “Task management” (Fig. 2)*.* The elements that were considered important in undergraduates and therefore created the basis to define the first dimension of AS-NTS are highlighted.

“Coordinating team members”, “communication” and “Leadership” were regarded as highly important in the focus group and performance could be observed in different levels during the field observation, therefore these elements created the basis for dimension two of ANAESTHESIOLOGY STUDENTS’ NON-TECHNICAL SKILLS: “*Teamwork and leadership”*.

Leadership, defined as the skill of directing others, coordinating, managing workload and motivating others [37] is often separated into two independent dimensions allowing for the assessment of different leadership styles [60] distinguishing between task orientation and team orientation. In this leadership model, “Task orientation” is closely related to our first two AS-NTS dimensions, therefore we decided to add *“Team orientation”* as third and final dimension of the AS-NTS. “Teamwork and leadership” emphasizes the collaborative processes to perform a task, whereas “Team orientation” focuses on the collaborative processes to build a team.

**Fig. 3:**
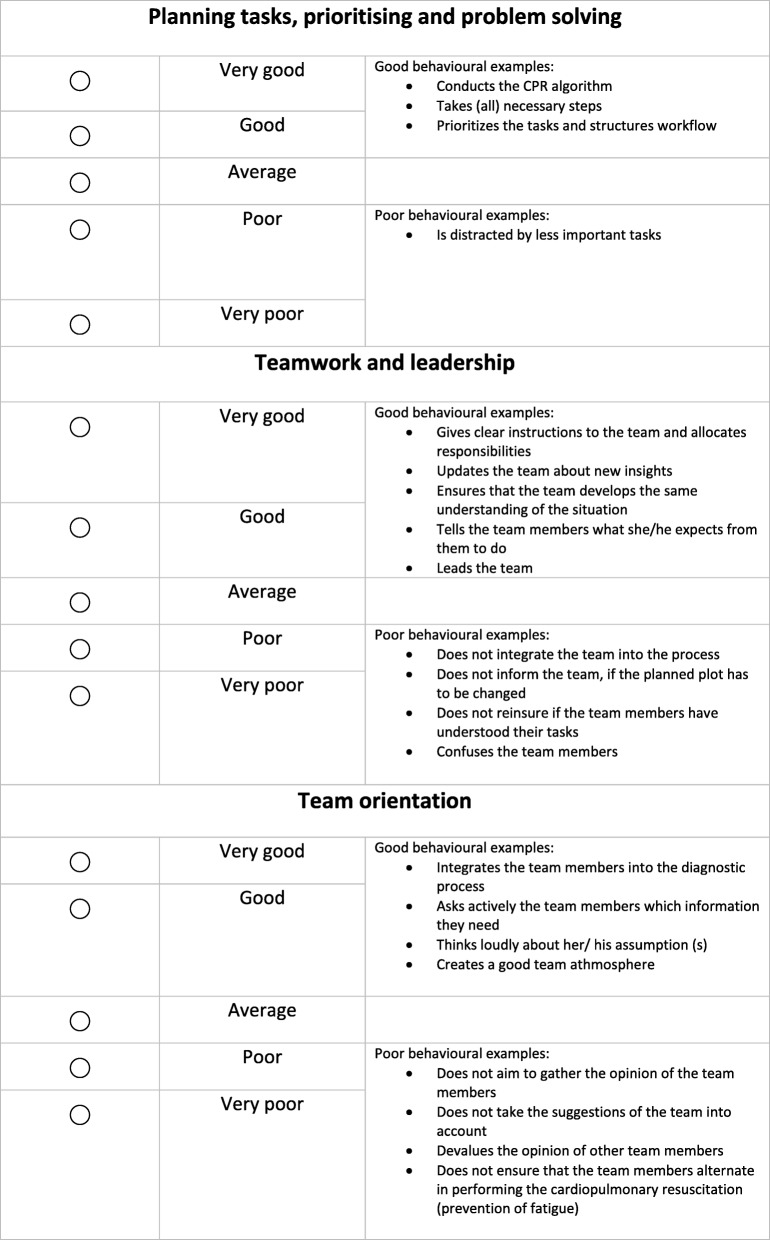
AS-NTS assessment tool (english version; the original German version has been added to the Additional file 1)

In contrast to the ANTS, performance is rated in the AS-NTS on the three dimensions and not on the level of skills. However, the underlying skill structure was used to give behaviorally anchored rating examples to clarify what a “good” or “poor” performance on each dimension might look like. In the final AS-NTS assessment tool (Fig. 3), a five-point Likert scale was used for each dimension, although the ANTS system has a four-point scale [32]. Cook et al. could show that, in regard to reliability and interrater reliability, there are no differences in 5- and 9- point scales in mini-clinical evaluation exercise [61].

### Feasibility and content validity of the scoring system

The interviews with eight anaesthesiologists in their first year of residency, who used both the AS-NTS and ANTS in simulation training (including video tapings), showed that no further dimension had to be added to the AS-NTS rating tool (step 4). Furthermore, they confirmed the feasibility of AS-NTS and concluded that in undergraduates, as well as in the first 2 years of residency in anaesthesiology, the ANTS system is too complex.

Without video tapings it is nearly impossible to complete ANTS, due to time shortness. This was already pointed out by the developers [5, 32]. The eight anaesthesiologists discovered the rating of the videos to be very time consuming and delaying the feedback loop.

These anaesthesiology trainees decided to continue their postgraduate training curriculum using AS-NTS, rather than ANTS, for their first 2 years of residency.

The results from an additional evaluation questionnaire, completed by 21 anaesthetits, who had used the rating tool at least three times in undergraduate medical education, confirmed that the AS-NTS was feasible and practical (Additional file 1: Table S1). Additionally, they rated the importance of each dimension of AS-NTS.

The content validity index for each dimension was calculated, reflecting the proportion of relevance [62]. The calculated content validity index for the first dimension of AS-NTS was 0.9, for the second dimension 0.95 and for the third dimension 0.8. A content validity index of 0.75 or higher is considered as “excellent” [33].

### Interrater reliability

The interrater reliability reached high levels of agreement (Table 6), except for dimension two, in the group of 3rd vs. 5th year residents (fair correlation). The ICC indicated a high rater agreement regardless of educational experience, training in anaesthesiology or familiarity with the AS-NTS rating tool.

**Table 6:** Interrater reliability of the six pairs of raters and of all data (98 rated simulation scenarios)

Raters	ICC D.1	ICC D.2	ICC D.3	ICC Overall	Cohen’s Kappa D.1	Cohen’s Kappa D.2	Cohen’s Kappa D.3	Cohen’s Kappa Overall
Interrater reliability of six pair of raters (67 emergency and anaesthesiology simulation scenarios)								
R1/R2	0.925	0.955	0.882	0.922	0.92	0.95	0.90	0.92
R1/R3	0.897	0.856	0.945	0.886	0.89	0.87	0.78	0.88
R1/R4	0.855	0.825	0.874	0.837	0.833	0.853	0.854	0.837
R1/R5	0.943	0.949	1	0.976	1	0.87	0.78	0.88
R1/R6	0.905	0.978	0.943	0.945	0.893	0.98	0.94	0.94
R7/R8	0.706	0.706	0.706	0.681	0.67	0.67	0.67	0.56
Interrater reliability of the 67 emergency simulation scenarios after data aggregation across raters’anaesthesiology training								
A./2nd year	0.880	0.893	0.830	0.868	0.87	0.8	0.82	0.87
A./3rd year	0.915	0.900	0.950	0.917	0.90	0.89	0.95	0.92
A./4th year	0.871	0.937	0.884	0,897	0.91	0.96	0.88	0.86
A./5th year	0.811	0.787	0.834	0.805	0.85	0.77	0.87	0.78
4th/3rd year	0.729	0.767	0.722	0.733	0.77	0.75	0.71	0.74
5th/3rd year	0.650	0.500	0.680	0.624	0.73	0.48	0.68	0.63
Interrater reliability of the 31 OR simulation scenarios with 3 raters per each simulation scenario								
ICC Dimension one		ICC Dimension two			ICC Dimension three		ICC overall	
0.737		0.780			0.684		0.738	

Abbreviations: *A.* Attending, *R.*: Rater, *Year* Year of anaesthesiology training

## Discussion

The development of the AS-NTS was performed in a stepwise approach, beginning with a review of pre-existing literature, continuing with focus group analysis and field observation, and ending with implementation and validation.

The steps were processed by means of empirical and qualitative research methods, which have gained a broad application in medical research [63–71].

During the field observations some skills proved to be difficult to observe and excluded from ANAESTHESIOLOGY STUDENTS’ NON-TECHNICAL SKILLS, based on developmental guidelines of assessment tools described by Abell et al., who recommend items to be excluded, if they are not observable in at least 50% of field observations [72].

Nonetheless, the excluded skills are part of most existing NTS taxonomies and regarding the importance of these skills, one might argue that they should still be taught and addressed in undergraduate education.

However, acquiring and refining NTS is an individual and ongoing process [73]. Therefore, in undergraduate training pre-cursors of some NTS should be assessed and evaluated. Further, most of the taxonomies from which the NTS list was extracted, are developed for postgraduate training- focussing on specialist level, which makes these skills not one to one transferable to undergraduates.

Those skills should be focused in more advanced educational levels, mostly in postgraduate training. Nevertheless, the aim of the study was to include as many NTS as possible into the ANAESTHESIOLOGY STUDENTS’ NON-TECHNICAL SKILLS, in order to assess them in undergraduates to provide accurate feedback, enhancing the learning process. [74] For this goal, skills were redefined during the development of ANAESTHESIOLOGY STUDENTS’ NON-TECHNICAL SKILLS, symbiosing some pre-defined NTS and focusing more on pre-cursors and underlying elements of skills. This adaptation process was not solely based on the expert- and focus groups- but was supported by literature and resulted in the new dimensions of ANAESTHESIOLOGY STUDENTS’ NON-TECHNICAL SKILLS, specifically designed for undergraduates. The adaptation step was necessary, as some NTS are highly important but not fully developed in undergraduates.

Transferred to the first dimension of ANAESTHESIOLOGY STUDENTS’ NON-TECHNICAL SKILLS, two main dimensions of described NTS (“Decision making” and “Task management”) were symbiosed to the first AS-NTS dimension *“Planning tasks, prioritizing and problem solving”.*

This might lead to the assumption a specific assessment of these skills is not possible, as they are assessed in the same dimension of performance and in pre-existing rating tools, they are separately assessed.

This objection can be warded by focusing on the developmental rational and existing literature:

“Decision making” is a complex skill which is divided into subskills and rated separately by some behavioral taxonomies [28, 29, 32]. Flowerdew et al. pointed out that it is not only making the decision which is of great importance, but also following the effects caused by the decision, like planning and prioritizing tasks to conduct the decision [38]. Here, “Decision making” is directly linked to the dimension “Task management”, which includes the elements: “Planning and preparing, prioritizing, providing and maintaining standard, identifying and utilizing resources”. Conducting the elements of “Task management” is the following consequence after “a decision is made”.

The comprehensiveness of “Decision making” and “Task management” regarding the training level of undergraduates was pointed out repeatedly, leading to the exclusion of these dimensions and focusing on some elements of these skills.

The elements of “Decision making” are: Identifying options, balancing risks, selecting options and re-evaluating [7, 20, 32, 56, 75].

Identifying options is necessary to solve a problem- in the decision making loop the risks and benefits of the solving strategy are re-evaluated – this concept of decision making is applicable in more complex scenarios than in undergraduate simulation training. Therefore, the focus group discussed and agreed to include “problem solving” as a less complex proxy of decision making into the first dimension of AS-NTS.

The strength of this study was scrutinizing the interrater reliability from different viewpoints. The interrater reliability was not only defined by a few designated raters, as in classical approaches. A two-step approach was chosen to analyse rater agreement, simultaneously examining if personal background (a.e. year of anaesthesiology training or experience in medical education) might influence ratings. First, agreement of rater pairs were analysed with a sufficient number of ratings, excluding agreement by chance, then data aggregation of the full sample was conducted based on anaesthesiology training, to calculate the interrater reliability. AS-NTS achieved excellent Interrater reliability, only within the group of 5th year vs. 3rd year anaesthesiology residents, the ICC and Cohens Kappa were “good” and only “fair” for dimension two of AS-NTS.

Data aggregation in the full sample, supports the result that the rating agreement is detached from anaesthesiology training and experience in medical education, fostering the usability of AS-NTS.

The strong involvement of *Rater 1* in the assessment of the interrater reliability might lead to the assumption one rater could influence all the other raters. Regarding our results, this is not the case, as data aggregation across all raters, in which Rater 1 is not represented predominantly, showed high agreement on ratings as well.

The dimensions of the final version of AS-NTS achieved excellent content validity indexes according to a guideline for evaluating standardized assessment instruments [35]. However, one weakness of the study is that the calculation of the content validity index was only calculated from the evaluation of twenty-one anaesthesiologists. Although there is no predefined sample size required to establish content validity [76], the effect of agreement by chance is higher in a small sample.

The AS-NTS has high potential to improve NTS assessment in undergraduate education and ultimately patient safety, because a lack of NTS leads to adverse events in high-risk settings [5]. A recent study by Hagemann et al. showed that NTS in undergraduate students are improved after only one seminar [21]. Due to its good feasibility, the AS-NTS could be applied to all students as a standardised assessment and feedback tool.

### Limitations

The AS-NTS has only been tested in German language at one institution with a limited number of teachers. Further studies should be conducted to establish the validity, reliability and feasibility of the English version.

## Conclusion

AS-NTS provides a structured approach to the assessment of NTS in undergraduates, providing accurate feedback. The findings of usability, validity and reliability indicate that the AS-NTS can be used by anaesthesiologists in different year of postgraduate training, even with little experience in medical education.

## Additional file

Additional file 1: AS-NTS. (DOCX 93 kb)

## Data Availability

The datasets used and/or analysed during the current study are available from the corresponding author on reasonable request.

## Abbreviations

ANTS: Anaesthesiology Non-Technical Skills
AS-NTS: Anaesthesiology Students’Non-Technical Skills
CVI: Content Validity Index
ICC: Intraclass correlation
NTS: Non-Technical skills
TS: Technical skills
