# Supplementary material for: Anaesthesiology students’ Non-Technical skills: development and evaluation of a behavioural marker system for students (AS-NTS)
Source: BMC Med Educ. 2019 Jun 13;19:205. doi: 10.1186/s12909-019-1609-8 (PMC6567593; doi:10.1186/s12909-019-1609-8)
Supplement: Supplementary file 1 — AS-NTS. (DOCX 93 kb) [file 12909_2019_1609_MOESM1_ESM.docx]

**Additional files / Supplementary**

**Developmental and validation steps of AS-NTS**

***Development and implementation of AS-NTS***

***Step 1: Review of published literature***

The expert group conducted a broad literature search reflecting upon NTS. This step was complemented by a comprehensive review of existing behavioural frameworks and taxonomies. Taking the results of both steps into consideration, a list of NTS, mainly relevant for emergency care settings was compiled and the NTS were discussed by the expert group, pointing out the relevance and importance of each NTS for undergraduates.

***Step 2: Focus groups and half-structured interviews for the development of AS-NTS***

Focus group discussions were held, in which every pre-defined NTS was analysed regarding its importance for undergraduates, taking into account that some skills can be trained and refined in postgraduate training. In a subsequent discussion a hierarchy of the NTS was specified.

Two experienced anaesthetic co-workers were also interviewed in half-structured interviews to include an interprofessional point of view.

In the focus groups, as well as in the half-structured interviews, open ended questions were used to facilitate the discussion concerning the NTS. Two questions were addressed for each NTS: (1) Is it required in undergraduate students and (2) to which extent can the skill be expected to be possessed by the student?

***Step 3: Field observation***

In a next step, field observations of teaching sessions in emergency medicine and anaesthesiology were performed to evaluate whether the defined skills could be observed and rated in undergraduate students.

In a pilot study of 20 simulation scenarios, the rating tool was tested for feasibility and practicality.

***Step 4: Validation***

***Questionnaire and focus group to investigate the content validity and feasibility of AS-NTS***

Eight first year anaesthesiology trainees, who participated in a postgraduate training curriculum of our department, which includes various training sessions which include simulation as one essential pillar, were introduced and trained in the ANTS and the new AS-NTS assessment tool. They used both tools more than five times each, in simulation training sessions of the curriculum. Afterwards they were asked if they could observe relevant NTS that were not captured by the AS-NTS rating tool, or if they could suggest other improvements to the AS-NTS.

For matters of content validity and feasibility, twenty-one anaesthesiologists in different stages of their residency, some of them with great- and some with minor experience in medical education, were aligned undergraduate training sessions of our department as medical educators, during the study period. Each of the twenty-one medical educators used AS-NTS during the training sessions for the calculation of the ICC, at least three times. Afterwards they answered a questionnaire on AS-NTS and rated the importance of each dimension of AS-NTS, which was the basis for the calculation of the content validity index.

*Supplement Table 1.: Questionnaire about AS-NTS.*

| **Question** |  | |
| --- | --- | --- |
|  | **yes** | **No** |
| Is the tool feasible | 87,5% | 12,5% |
| Did you have any additional effort or expenditure of time while using AS-NTS | None | 100% |
| Do you think the dimensions for NTS regarding students is sufficient? | 100% | 0% |
| Would you use the AS-NTS further on as a feedback tool? | 87,5% | One colleague stated that, independently from the tool or any tool rating NTS is very hard for him |

| **Aufgaben planen, priorisieren und Problemlösung** | | | |
| --- | --- | --- | --- |
|  | ⃝ | sehr gut | gute Verhaltensbeispiele:   - hält sich an den erlernten Wiederbelebungs-algorithmus - die erforderlichen Maßnahmen werden eingeleitet - priorisiert wichtige Aufgaben - strukturiert den Arbeitsablauf |
|  | ⃝ | gut |  |
|  | ⃝ | durchschnittlich |  |
|  | ⃝ | schlecht | schlechte Verhaltensbeispiele:   - lässt sich von weniger wichtigen Aufgaben ablenken |
|  | ⃝ | sehr schlecht |  |

| **Teamwork und Führung** | | | |
| --- | --- | --- | --- |
|  | ⃝ | sehr gut | gute Verhaltensbeispiele:   - gibt den Teammitgliedern klare Anweisungen und verteilt die verschiedenen Aufgaben - bringt das Team regelmäßig auf den neusten Stand - stellt sicher, dass die Teammitglieder ein gemeinsames Verständnis vom Problem entwickeln - sagt den Teammitglieder, was sie/er von ihnen erwartet - übernimmt aktiv die Führung im Team |
|  | ⃝ | gut |  |
|  | ⃝ | durchschnittlich |  |
|  | ⃝ | schlecht | schlechte Verhaltensbeispiele:   - bindet die Teammitglieder nicht ein - informiert das Team nicht, wenn vom bisher geplanten Handlungsablauf abgewichen werden muss - versichert sich nicht rück, dass die Teammitglieder ihre Aufgaben verstanden haben und diese erfüllen - verwirrt oder irritiert die Teammitglieder |
|  | ⃝ | sehr schlecht |  |

| **Teamorientierung** | | | |
| --- | --- | --- | --- |
|  | ⃝ | sehr gut | gute Verhaltensbeispiele:   - bindet die Teammitglieder in den diagnostischen Prozess mit ein - fragt die Teammitglieder aktiv nach benötigten Informationen - denkt laut über seine Vermutungen nach - sorgt für eine angenehme Atmosphäre im Team |
|  | ⃝ | gut |  |
|  | ⃝ | durchschnittlich |  |
|  | ⃝ | schlecht | schlechte Verhaltensbeispiele:   - holt keine Meinungen der anderen Teammitglieder ein - berücksichtig deren Vorschläge nicht - wertet die Meinung eines Teammitglieds ab - kümmert sich nicht darum, dass man sich bei der Herz-Druck-Massage abwechselt |
|  | ⃝ | sehr schlecht |  |

*Supplement Figure 1. German version of AS-NTS.*

*The simulation scenarios of the emergency training sessions*

The students roleplay an emergency team that is dispatched to a scene. Prior to their arrival, they receive a short briefing of why the emergency service has been alerted, e.g. acute chest pain. The goals are to establish basic monitoring, and follow the ABCDE algorithm. Therapeutic options have to be taken into consideration and be implemented (for example, inhaled beta-agonists in an acute asthma exacerbation).

When cardiopulmonary arrest occurs, resuscitation has to be performed, taking into account the ACLS algorithms.

*ACLS I*: The students are called to an unconscious patient. The patient presents with either an asystole or a ventricular fibrillation.

*ACLS II:*

*Hyperkalaemia*

The students are called to a dialysis practice, where a patient has chest pain. He has missed his dialyses appointment from the previous day. After 90 seconds, he suffers a cardiac arrest. The students have to establish the basic monitoring, perform ACLS, consider what caused the cardiopulmonary arrest, and consider which further steps should be taken.

*Hypothermia*

The students are called to the central station where a homeless person has been found, unresponsive. The patient presents with an extreme bradycardia and ventricular extrasystoles due to hypothermia. The only response is on pain stimulation. The students have to establish the basic monitoring, as well as consider therapeutic options. After 90 seconds, the patient converts to a ventricular fibrillation. The students than have to perform ACLS and to think further about the primary cause for the cardiopulmonary arrest.

*Aspiration*

The students are called to Mr. Meyer, who suddenly coughed forcefully during dinner and then collapsed. The patient presents in asystole. He has aspirated a piece of meat into his pharynx, which is seen when laryngoscopy is performed.

*ACLS III:*

*Pneumothorax*

The students are called to a high school, where an 18 year-old scholar is complaining about chest pain. When the team arrives, he has dyspnea and complains of intense chest pain. After 90 seconds, he converts to an asystole due to a tension pneumothorax. In addition to following the ACLS algorithms, the students have to think about the cause and to treat the tension pneumothorax.

*Acute coronary syndrome*

The students are called to a company, where a worker is complaining of chest pain, resulting in the ambulance being called.

He presents with ST-elevation on the ECG (if students succeed in establishing the basic monitoring within 90 seconds). After 90 seconds, he converts to ventricular fibrillation.

*Pulmonary embolism*

The students are called to the airport, where a businessman is complaining of chest pain shortly after exiting the aircraft (New York JFK to Hamburg-Helmut Schmidt). The patient presents with tachycardia and hypotension (if the students succeed in establishing the basic monitoring within 90 seconds). After 90 seconds, he converts to asystole due to a PE.

*Asthma*

The students are called to a high school, where a 17 year-old girl with a history of asthma presents with extreme shortness of breath. If the students ask, she has already used her asthma sprays several times. There are marked expiratory wheezes in both lungs on auscultation with a stethoscope. Her first oxygen saturation is 67%, not responding much to oxygen therapy (if students succeed in establishing the basic monitoring within 90 seconds). After 90 seconds, the oxygen saturation sinks progressively until the girl converts to asystole.

*AV-Block*

The students are called to the surgical ward, where a post-gastrectomy Patient has collapsed in the bathroom. Upon arrival, the patient is conscious. The ECG shows an intermittent AV-Block III (if the students succeed in establishing the basic monitoring within 90 seconds). After 90 seconds, the patient becomes unconscious due to the AV-Block III.

*The simulation scenarios of the anaesthesiology (OR) training sessions*

The students roleplay an anaesthesiology team that is dispatched to a scene. Prior to their arrival, they receive a short briefing on how the setting of the simulation scenario is, e.g. “you take over a narcotized patient from your colleague”.

During each simulation scenario incidents occur, which have to be handled by the anaesthesiology team. Each action of the anaesthesiology team is shown by physiological responses of the patient (e.g. fluid administration increases blood pressure).

Therapeutic options have to be taken into consideration and be implemented.

*Inadequate depth of anaesthesia*

The students are asked to induct anaesthesia in a 26 year-old patient for cholecystectomy. After induction, the patient presents with tachycardia and hypertension, due to inadequate depth of anaesthesia.

*Hypovolaemia*

The students are asked to perform general anaesthesia in an 82 year-old patient with a femur fracture which has to be fixed. The patient is confused, so that adequate anamnesis is not possible. After anaesthesia induction, the patient presents with severe tachycardia and hypotension, due to hypovolaemia.

*Acute coronary syndrome*

The students take over a patient in general anaesthesia for a femur fracture. Shortly after takeover the patient presents ST-elevations on the ECG.
